# Let Them Talk: Coping with PrEP-Related Stigma and Sustaining PrEP Persistence Among Men Who Have Sex with Men in Tanga, Tanzania

**DOI:** 10.3390/healthcare14020259

**Published:** 2026-01-21

**Authors:** Faithness Kiondo, Emmy Metta, Elia John Mmbaga, Kåre Moen, Calvin Swai, Melkzedeck Leshabari

**Affiliations:** 1 Department of Behavioural Sciences, Muhimbili University of Health and Allied Sciences, Dar-es-Salaam 11103, Tanzania; 2 Department of Community Medicine and Global Health, University of Oslo, 0450 Oslo, Norwaykare.moen@medisin.uio.no (K.M.); 3 Department of Educational Psychology and Curriculum Studies, University of Dodoma, Dodoma 41218, Tanzania

**Keywords:** pre exposure prophylaxis, stigma, men who have sex with men, persistence, HIV/AIDS

## Abstract

**Background:** Pre-exposure prophylaxis (PrEP) offers over 99% protection against HIV when used consistently, but stigma continues to undermine persistence in care. While much research has described the external manifestations of PrEP-related stigma, less is known about how individuals cope with these stigmas and how such coping processes influence persistence. Guided by Social Cognitive Theory, this study examined the psychosocial strategies men who have sex with men (MSM) in Tanzania use to cope with PrEP-related stigma and sustain persistence in care. **Methods**: Thirty-two in-depth interviews were conducted with purposefully selected MSM aged 18–38 years at Ngamiani Health Centre in Tanga region. The sampling included both persistent and non-persistent PrEP users with variation in age and sexual position preferences. Participants were sampled for variation in persistence status (persistent and non-persistent), age, and sexual position preference to capture heterogeneity in stigma experiences and coping processes. Interviews were conducted in Kiswahili, audio-recorded, transcribed, translated, and analyzed using reflexive thematic analysis. **Results**: Participants described PrEP-related stigma as socially constructed through narratives that equated PrEP with HIV treatment, labeled it a “gay pill,” associated it with promiscuity, or linked it to bodily harm or increased HIV risk. These stigmas impact persistence in care through discouraging clinic visits and daily pill taking. However, some participants remained persistent in care despite stigma by using protective mental strategies such as personal agency, mental time travel, and affirmation from supportive social connections, which buffered emotional impacts and sustained persistence. **Conclusions**: Persistence in PrEP care is shaped not only by stigma in the social environment but also by how individuals interpret and respond to it. Interventions should therefore combine structural stigma-reduction efforts with mental health-informed strategies that strengthen agency and supportive social relationships to sustain PrEP engagement among MSM.

## 1. Introduction

HIV prevention and treatment have advanced considerably in recent decades, leading to substantial reductions in new HIV infections. Despite this progress, HIV remains a major public health challenge among key populations, particularly men who have sex with men (MSM), sex workers, and people who use drugs [1,2,3]. At the end of 2024, an estimated 40.8 million people were living with HIV globally, and approximately 630,000 deaths were attributed to AIDS-related illnesses [1]. This burden is not evenly distributed and is shaped by social and structural factors such as stigma, discrimination, and criminalization, rather than biomedical risk alone [4,5,6,7].

Understanding engagement with HIV prevention therefore requires attention not only to social conditions but also to how individuals cognitively interpret and respond to those conditions. Social Cognitive Theory (SCT), proposed by Bandura, offers a useful framework for this purpose by conceptualizing health behaviour as the product of reciprocal interactions between environmental influences, cognitive processes, and individual action [8]. This perspective is particularly relevant for prevention behaviours such as Pre-exposure prophylaxis (PrEP) persistence, which require sustained personal motivation in the context of stigmatizing social environments.

PrEP represents one of the most effective biomedical tools for HIV prevention, reducing the risk of HIV acquisition by more than 99 percent when used consistently [9,10,11]. For MSM, sustaining the PrEP persistence is especially important given the high concentration of HIV risk within their sexual networks [1,2,3].

In this study, persistence is defined as continued PrEP use alongside regular attendance at clinic visits for refills and related services [12,13,14]. While persistence may appear straightforward in theory, in practice it is often undermined by stigma attached to PrEP use, which shapes how the intervention is socially understood and personally experienced [15,16,17,18]. Rather than being interpreted solely as a protective health behaviour, PrEP use is frequently viewed through narratives of suspicion, moral judgment, and social labeling [15,16]. Following Link and Phelan [19], stigma is understood here as a social process involving labeling, stereotyping, separation, status loss, and discrimination.

For MSM, PrEP-related stigma is layered onto existing stigma related to sexuality, morality, and perceptions of sexual deviance, particularly in settings where same-sex practices remain criminalized, such as Tanzania and much of sub-Saharan Africa [17,19,20,21]. Studies across diverse contexts have consistently shown that such stigma negatively affects PrEP uptake and persistence, discouraging clinic attendance and adherence while increasing HIV vulnerability [15,16,17,18].

Although the external and social manifestations of PrEP-related stigma are increasingly well documented, less is known about how MSM cognitively interpret these stigmatizing experiences and how such interpretations shape decisions to persist or disengage from PrEP care. Emerging scholarship suggests that stigma operates not only at the social level but also through internal psychological processes that shape identity, motivation, and health-related decision-making [22,23]. Yet, most PrEP research continues to emphasize community-level stigma, leaving the internal meaning-making processes emphasized by Social Cognitive Theory underexplored.

Guided by Social Cognitive Theory, this study examines how MSM in Tanzania cognitively interpret PrEP-related stigma and how these interpretations interact with social contexts to influence persistence in PrEP care. By foregrounding lived experiences, this study conceptualizes stigma and its implications as both an external social force and an internal process of meaning-making that shapes persistence in PrEP services. Accordingly, this study addresses the following research question: How do men who have sex with men in Tanga, Tanzania experience and cope with PrEP-related stigma, and how do these experiences influence sustained PrEP persistence?

## 2. Materials and Methods

### 2.1. Design

We employed a qualitative design to explore how men who have sex with men experience, interpret, and respond to stigma related to PrEP persistence. The qualitative approach was chosen for its ability to capture the depth, complexity, and meaning of lived experiences that cannot be adequately understood through quantitative approaches [24].

### 2.2. Participants and Sampling

Purposive sampling seeking broad variation in age and sexual position preference was used to recruit eligible participants from two subgroups (persistent and non-persistent). Eligible participants were men who have sex with men aged 18 years and older, residing in Tanga region, who had initiated PrEP at Ngamiani Health Centre at least six months before the interview period. Persistent participants were those who attended their follow-up visit within 28 days of their scheduled appointment, while non-persistent participants were those labelled as disengaged in the hospital register, meaning they had not attended the clinic for more than two months after their scheduled visit even after reminders from peer educators or clinic staff. Furthermore, we sampled for variation in age and sexual position preference to capture heterogeneity within the MSM community, as these characteristics correspond to distinct social roles, relational dynamics, and stigma exposures that can shape varied patterns of PrEP persistence. Peer educators identified eligible clients from the clinic register and contacted them outside clinical encounters to invite their participation. Information on participants’ persistence status was obtained from clinic records, while details on their sexual position preference (insertive, receptive, or versatile) were drawn from peer educators’ existing knowledge of community networks and later confirmed during interviews. As members of these networks, peer educators were familiar with participants’ self-identified roles, which helped ensure diverse representation across these characteristics. A total of 32 participants were interviewed, including 14 persistent and 18 non-persistent individuals. Recruitment stopped when meaning saturation was reached, indicated by repeated concepts across interviews and the absence of new insights or thematic dimensions despite continued sampling across MSM subgroups.

### 2.3. Ethical Considerations

This study received ethical approval from Muhimbili University of Health and Allied Sciences Institutional Review Board (MUHAS-REC-08-2024-2409). We also obtained permission from Tanga City Council, the Tanga Regional Medical Officer and from Ngamiani health facility. All participants were informed about this study’s aims, procedures, risks, and benefits and they were informed that they could withdraw at any time without repercussions. All participants provided informed consent for their participation and audio recording of the interviews. To maintain confidentiality, participants were identified by numbers, the audio files were stored in password-protected devices, and the transcripts were anonymized to remove all the identifiable information. No separate list linking identities to numbers was created; numerical codes were assigned directly through the audio-file naming system (for example, the first interview became Participant 1), ensuring that no document existed that could connect personal identities to the anonymized data. Interviews were conducted in private spaces within the health facility to ensure privacy and minimize risk.

### 2.4. Data Collection

Data were collected through in-depth face-to-face interviews conducted in Kiswahili which is the native language for both the interviewer and the participants. A semi-structured interview guide featuring open-ended question, piloted with two men who have sex with men not included in this study to ensure clarity and cultural relevance was used. The interviews were conducted by the first author, a female psychologist with training in qualitative interviewing and experience working with key populations. Rapport was built with the help of peer educators who introduced participants and reassured them about confidentiality. Each interview lasted about 45 to 60 min and was audio-recorded with participants consent. During the interview, the interviewer remained reflexive about how her gender and professional background might shape interactional dynamics and used a neutral, non-judgmental approach to support participant comfort and disclosure. The researcher also took field notes after each interview to document contextual observations. Interviews took place in private rooms with no other individuals present. Data collection occurred between February to May 2025.

### 2.5. Data Analysis

Data were analyzed using reflexive thematic analysis [25], which views analysis as an active and interpretive process where researcher subjectivity is regarded as a resource rather than a bias. Audio recordings were transcribed in Kiswahili and translated into English by a bilingual researcher experienced in qualitative methods. To reduce meaning loss, each translation was checked against the original Kiswahili transcript for accuracy and nuance, and a second bilingual reviewer examined a subset to confirm consistency in interpretation Inductive coding was then performed to capture both explicit and latent meanings within the data. Codes were iteratively refined and grouped into broader themes which were then further reviewed to ensure they reflected patterns across the dataset. For example, within the theme PrEP care as HIV care, codes such as “pills look like ARVs,” “clinic located in HIV section,” “fear of being seen at the HIV clinic,” “carrying pills attracts gossip,” and “hiding pills to avoid being mistaken for HIV positive” were merged into the theme PrEP care as HIV care. Social Cognitive Theory served as a sensitizing framework during theme interpretation, where its core constructs guided sense-making without influencing code generation or predetermining analytic outcomes.

### 2.6. Quality and Rigor

We addressed trustworthiness following Lincoln and Guba’s criteria [26], which include credibility, transferability, dependability, and confirmability. Credibility refers to the confidence in the truth of the data and interpretations, which was enhanced through prolonged engagement, where the first author conducted, transcribed, read, and translated all interviews, allowing for immersion in both content and context. Analytic memos and peer debriefing dialogues were employed to challenge assumptions, explore alternative perspectives, and strengthen analytic coherence. Emerging themes were discussed and validated during regular debriefing sessions with two qualitative researchers who did not participate in data collection, ensuring intersubjective verification and minimizing single-researcher bias. Transferability concerns how well findings apply other settings, supported here by providing thick descriptions of the study setting, participant characteristics, and the cultural context of PrEP use in Tanzania. Dependability relates to the stability and consistency of the research process, which was ensured through a detailed documentation of the analytic steps and repeated analysis of individual transcripts and the entire dataset. Confirmability refers to the extent to which findings are driven by participants rather than researcher bias, reinforced through reflexive journaling and a transparent audit trail documenting analytic decisions from coding to theme development.

## 3. Results

This section presents the findings from the interviews, beginning with the characteristics of the participants and then moving to the themes unfolded. The themes are organised to reflect the different layers that emerged across the dataset: starting with the external stigma and misinterpretations surrounding PrEP, then moving to the internal processes participants used to navigate these pressures, and finally to the social support that helped reinforce their efforts.

### 3.1. Participants Characteristics

A total of 32 men who have sex with men participated in this study, with 18 currently not persistent in PrEP care and 14 persistent. Although the sample includes more non-persistent than persistent participants, this imbalance did not affect interpretation because the analysis sought meaning saturation rather than numerical balance, and both groups contributed sufficient depth for theme development. The majority were single (21), about a quarter were married to women (6) or cohabiting with male partners (5). Participants were mainly aged between 25 and 34 years (15), followed by 18–24 years (12). Educational backgrounds varied, with 15 having completed secondary education. Common occupations included motorcycle taxi ‘*bodaboda*’ drivers (9), small-scale traders (11), and students (6) as shown on Table 1.

### 3.2. Socially Constructed Stigma Around PrEP

Participants described community narratives that contributed to the stigmatization of PrEP users. These narratives emerged across both persistent and non-persistent participants, reflecting their shared exposure to and lived experiences of stigmatizing community discourses around PrEP. Five recurring narratives were evident.

#### 3.2.1. PrEP Care as a HIV Care

Among the narratives that were mentioned was that PrEP care was commonly misunderstood to be HIV care because of similarities in the appearance of the pill and how it is delivered within the healthcare system. The pills looked similar to antiretroviral treatments (ARVs) in the physically appearance, the container used, and even the sound the pills make when inside the container. Furthermore, participants noted that PrEP services were often provided in the same clinic and room where people living with HIV received their care, which further strengthened the connections between PrEP and HIV care. As a result, some participants avoided using the pills, carrying them or visiting the clinic altogether to prevent being seen with the pills and labelled as HIV positive.

“*These pills look like ARVs, so when someone sees you with them, they just assume you have HIV. And the clinic we go to is the same one used by people living with HIV. In Tanga everyone knows everyone, so when someone sees you there, they say you also have HIV. I know there’s a difference because when HIV clients collect their pills, they have to scan their fingerprints, but I don’t. Still, you can’t defend yourself once they’ve seen you; they already believe you have HIV and they go tell others that you have HIV.*”

(P 18)

#### 3.2.2. PrEP as a ‘Gay’ Pill

While the previous theme centred on health-related misclassification linked to HIV status, this theme reflects a different layer of identity concern rooted in sexuality and social role expectations. Participants explained that in their communities PrEP is usually called “*vidonge vya mashoga*” (pills for same-sex attracted men who are socially understood to be effeminate and to take the receptive role in sexual relations with men). This label immediately carries stigma, as being linked to homosexuality often causes shame, gossip, and social rejection. For some, the stigma not only made them reluctant to take the pills around others but also caused fear of being seen going to PrEP clinics. Among MSM who identify as male in the community, the label *shoga* was especially harmful. In local use, *shoga* refers to men who take the receptive role during anal sex, a role often seen as feminizing and deeply shameful compared to the insertive role which is viewed as manly. As a result, using PrEP became a visible sign one’s sexual role, increasing stigma and heightening fears of being publicly identified. This fear went beyond just taking the pills and directly shaped participants’ willingness to remain persistent in PrEP services.

“*Sister, you should know that out here in the streets, people call these pills pills for ‘mashoga’ and prostitutes. So the moment someone sees you with them, they automatically assume you’re the one being penetrated by other manly guys. For us who share a room in a ghetto, it becomes really hard to even use them… where would you even keep the pills? The moment your boys see them, the news spreads that you are being penetrated*”

(P7)

#### 3.2.3. PrEP as a Marker of Promiscuity and Sexual Recklessness

Another aspect that came up in interviews was that PrEP use was taken to be a marker of promiscuity and sexual recklessness. This perception stemmed from the main question asked, “*why do you need protection from HIV if you are only with one person?*”. In this context carrying pills or visiting PrEP clinics was seen as signaling engagement with multiple or risky partners instead of health precaution. These narratives spilled over into intimate relationships, where PrEP use was interpreted as a sign of infidelity and became a source of questioning, conflict, and mistrust. For some participants, pressure to show loyalty or avoid conflict led to concealment, missed refills, or stopping PrEP altogether.

“*You can have your main partner, someone who’s really taking care of you, and when they see the pills they say, ‘I’m with you alone so why are you taking these pills? If it’s just me and you, or do you have other men out there and you are just using me for your personal gains?’*”

(P3)

#### 3.2.4. PrEP as a Magnet for HIV

Another belief described by participants was that the PrEP pill acted as a magnet that attracts HIV. This belief was rooted in community stories suggesting that prolonged PrEP use alters the body’s natural immunity and makes it dependent on PrEP to stay protected from HIV. Consequently when PrEP is discontinued, the body purportedly “pulls” HIV closer, increasing the persons vulnerability compared to someone who never used PrEP. Although medically incorrect, this reasoning was emotionally charged and centered around risk comparison, people who had never taken PrEP were believed to be more resistant to HIV than those who had used it and stopped, and the longer PrEP was used, the more vulnerable the body was thought to become after discontinuation.

“*I’ve been on PrEP for a year, but the way people talk is that if I stop now and get exposed to HIV, I’ll get it more easily than someone who never took the pills. It’s like these pills have some kind of chemical that attracts HIV. So if you plan to quit, you better quit early. But if you continue, then you just have to stick with it, no turning back…*”

(P24)

#### 3.2.5. PrEP as a Pill That Harms Bodily Organs

Participants also explained that PrEP was believed to damage internal organs especially with long term use. These beliefs were not based on personal experience with side effects, but rather on assumptions or hearsay. The organs reportedly thought to be harmed by PrEP included reproductive organs with one saying, “*They say if you take it for too long, it will make you permanently impotent*”(P,9), as well as the liver and kidneys, with another stating, “*If you take these pills for a long time, they can damage your kidneys or even your liver…*”(P17). These fears were strong enough to lead some participants to stop taking PrEP early. Although the participants who continued using PrEP did not fully believe these claims, the fear persisted, indicating that even misinformation that is not accepted as truth can still create uncertainty.

“*Me, I’m still taking them, but honestly sometimes you hear so many things… it makes you pause. Like, what if? Even if I don’t fully believe it, it stays in your mind*”

(P4)

### 3.3. Persistence Protective Mental Processes Amidst the Stigma

Although experiences of PrEP-related stigma were widely reported, participants responded to these experiences differently. While most had encountered similar stigmatizing narratives, their choices about continuing with PrEP varied. The analysis identified several protective meaning-making strategies that seemed to reduce the impacts of stigma and helped some participants stay engaged in care despite social pressures.

#### 3.3.1. Personal Agency

Personal agency, the inner ability to guide one’s thoughts, actions, and motivations based on personal beliefs and values emerged as a key in maintaining persistence in PrEP care despite the social noise around it. Agency showed participants’ capacity to remain true to their own understanding and to take responsibility for their health decisions, even when faced with stigma and community judgment. Several participants shared that persistence required a conscious mental attitude of focusing on their own reasons for using PrEP instead of being influenced by outside talk and gossip.

“*Everyone talks and each person says their own thing. But if you know why you’re using it you have to calm your mind and say ‘Let them talk and as for me, I’ll keep going, the choice is yours no one makes it for you…you just have to have a clear mind and not easily swayed*”

(P23)

#### 3.3.2. Mental Time Travel

Another key process that supported persistence in PrEP care was what participants described as mentally projecting themselves into the future. Many who stayed engaged in PrEP talked about imagining the kind of life they wanted and then making present-day decisions to help secure that future. Their continued PrEP use was not only motivated by fear of HIV but also by hope and the vision of a better life. Even when faced with stigma, the idea of protecting their future gave participants a strong reason to keep using PrEP every day. For example, one participant shared his dream of becoming a doctor and explained how PrEP helps secures that dream.

“*I want to become a doctor so I can give people education, But when you have HIV, you’re constantly worrying, what if people find out? You lose your peace. You start feeling like maybe you won’t achieve your dreams. But with PrEP, I know I’m protected. Every month I test, I check my health, and I feel strong. I’m using my pills, I’m okay and I’m happy. I feel joy all the time because I know my dreams are valid*”

(P6)

### 3.4. Affirming Social Connections as External Reinforcement

While much of what supported persistence originated from internal mental processes, participants also highlighted the importance of external reinforcements in maintaining and strengthening these internal capacities to continue with PrEP. Trusted friends and understanding healthcare providers acted as external reinforcements that helped alleviate the emotional burden of stigma and normalized PrEP use. These social connections offered encouragement and reassurance, boosting participants confidence that their choices to stay in care was justified. Although not a mental process itself, affirming support functioned by reinforcing participants’ inner resolve and safeguarding the mental strategies they relied on to persist in PrEP care.

“*For me, I have two people I told that I am using and they really encouraged me. They’d tell me ‘If you’ve decided to use the medications, then continue. If you keep thinking about what others are saying, your life will never move forward. Stand on your ground because you’re not a child anymore. If that is your decision, own it.’*”

(P14)

## 4. Discussion

PrEP has been widely described as a biomedical promise for HIV prevention; however, our findings show that this promise is yet to be fully realized because stigmatizing narratives and social responses have negatively shaped the way PrEP is understood and experienced. Rather than being seen solely as an empowering prevention tool, users of PrEP have been subjected to suspicion, moral judgment, and labeling. This whole experience is similar to what Goffman argued as the main task of stigma- that it transforms what could otherwise be a valued behavior into a “spoiled identity” [27]. Link and Phelan further explain how stigma becomes socially entrenched through labeling, stereotyping, separating “us” from “them,” and enforcing status loss and discrimination [18]. In our study these processes were evident in how PrEP use triggered assumptions of HIV infection, promiscuity or non-normative sexuality leading some participants to reduce adherence, avoid clinic refills, or disengage entirely. Such findings echo research across sub-Saharan Africa and beyond, which has consistently shown that PrEP-related stigma undermines uptake and retention [14,15,16,17].

What emerged as particularly notable in this study, was the cultural framing of the PrEP pill as the ‘*shoga*’ pill, where any male seen using PrEP was labelled as a *shoga*. *Shoga* is a term originating from Swahili speakers used to refer to female friendships and is used locally to feminize and stigmatize men who engage in receptive sex [28,29]. For men who wish to preserve their masculinity and keep their sexual practices private rather than public identities (closeted), this label posed a significant social identity threat. Concealment is widespread among sexual minorities in sub-Saharan Africa, with nearly 89.5% hiding their orientation [30,31], and in Tanzania closeted men strive to carefully orchestrate their ‘masculinity’ through clothing and heterosexual relations [29]. Therefore, being labelled a ‘*shoga*’ directly threatens their carefully curated masculine identity, leaving men using PrEP with no choice but to use it discreetly or disengage from the services altogether.

This pattern aligns with broader evidence from HIV services showing that closeted men are less likely to test for HIV, disclose sexual behaviour to providers, or engage consistently in prevention programmes [31,32], increasing both individual and population-level vulnerability. While earlier HIV and sexuality studies in sub-Saharan Africa have documented widespread concealment and lower engagement with PrEP services, much of this evidence has been quantitative and descriptive, demonstrating reduced uptake without explaining the underlying processes. This study extends that literature by offering qualitative insight into how existing practices of concealment intersect with the framing of PrEP as a ‘shoga pill’, intensifying fears of feminization and unwanted visibility and shaping decisions to disengage from PrEP services.

Alongside moral and social labeling, participants associated PrEP use with fears of physical harm, including beliefs that it damages internal organs such as the liver and kidneys, causes infertility, or increases vulnerability to HIV. These findings mirror results from other settings in Africa where participants expressed fears that PrEP could lead to long-term health damage [33,34,35]. These narratives did not exist in isolation; they reflected a broader social memory of mistrust toward new biomedical interventions. For instance, during the COVID-19 pandemic, many people hesitated to take vaccines because of fears about vaccine safety, including worries that the shots might damage organs and cause long-term health problems [36,37]. As much as such fears are often explained as ignorance in public health discussions, psychologically they reflect adaptive meaning-making in conditions of uncertainty and escalated perceived health threats. When information about new biomedical interventions is limited, individuals make sense of it through their own reasoning, conversations, and prior experiences to re-establish coherence and fill informational gaps. These broader reflections, however, are offered simply to situate the findings within a wider context and are not intended as direct representations of participants’ accounts.

In the specific case of PrEP, this process is intensified by the way the intervention has been positioned as a prevention tool for the so-called “key populations” in much of sub-Saharan Africa. National guidelines across several African countries explicitly describe PrEP as a service prioritized for groups such as men who have sex with men, sex workers, and people who inject drugs [38]. These groups are already heavily stigmatized, marginalized, and often criminalized within their own societies [39,40]. While programmatically this focus aims to reach those at highest risk, it also influences how the intervention is socially perceived. When prevention information mainly circulates within populations already rejected by the mainstream community, mistrust becomes an understandable psychological response. In such contexts, suspicion can easily develop into beliefs that “they want to harm us,” especially when broader community PrEP education is lacking. This highlights the urgent need for PrEP education rooted in transparency, community dialogues, and inclusion to demystify the intervention and foster informed understanding across all segments of society. Such an approach provides comprehensive and accurate information on how PrEP works and includes others who are at significant risk but fall outside the conventional definitions of key populations, ensuring full HIV prevention.

While experiences of stigma were linked to disengagement by some (former) PrEP users, this was not always the case, which helps explain the varied patterns of disengagement as many participants were exposed to the same stigmatizing narratives, yet their responses differed. For some, stigma cause them to withdraw from PrEP, while others found ways to persist in care despite the burden of stigma, a pattern consistent with other documented experiences [41]. These findings show that individual behavior is not solely driven by social circumstances but also by how people mentally process those circumstances, as Bandura argued in his theory of Social Cognitive [23]. In this theory, behavior is explained through reciprocal determinism, where personal factors, behavior, and the environment continually interact and influence each other. Within this framework, stigma acts as an environmental pressure, but its effect on engagement ultimately depends on the individual’s cognitive resources, which influence whether the individual internalizes, resists, or reinterprets the stigmatizing messages.

Participants identified three protective processes that support persistence in PrEP care: personal agency, future-oriented thinking, and affirming social connections. Personal agency closely aligns with the SCT construct of self-efficacy, reflecting individuals’ confidence in their ability to make and sustain health-protective decisions de-spite stigma. Personal agency closely mirrors PrEP self-efficacy, which has been shown across settings to predict adherence and persistence [42,43,44,45]. Future-oriented thinking corresponds to outcome expectancies within SCT, as participants’ decisions to persist were shaped by anticipated future health, stability, and life goals. Future orientation, seen in participants’ hopes and health goals, aligns with studies indicating that thinking about future outcomes predicts lower HIV risk and better adherence [46,47,48]. Affirming social connections reflect reciprocal determinism, whereby supportive social environments reinforce individual motivation and sustain health behaviours over time. Building supportive social relationships buffers the emotional impact of stigma, a finding aligning with evidence that social support helps with initiation and continued use [42,49,50,51]. Overall, these processes show that persistence in PrEP care involves not just overcoming external stigma but also the dynamic interaction between cognitive resources, anticipated outcomes, and reinforcing social environments, as described in Social Cognitive Theory. Generally, our findings emphasize the importance of individual meaning-making and supportive social resources in maintaining persistence in PrEP care. Interventions that address PrEP stigma at the community levels combined with efforts to boost people’s sense of agency and confidence could help men who have sex with men persist in PrEP care. This, in turn, may reduce new HIV infection rates and ultimately contribute to achieving the global goal of ending HIV as a public health threat by 2030.

## 5. Conclusions

This study highlights that PrEP persistence among MSM in Tanzania is fundamentally constrained by structural and social stigma that shapes how PrEP is understood and used. While individual cognitive resources and supportive social connections can buffer these pressures, they do not replace the need to address stigma within communities, health systems, and policy environments. Interventions should therefore prioritize structural stigma reduction, alongside mental-health-informed strategies that strengthen agency, future orientation, and affirming social support. Addressing both the social conditions that produce stigma and the resources that help individuals navigate them is essential for sustaining PrEP engagement and reducing HIV vulnerability among MSM. 

## Figures and Tables

**Table 1:** Distribution of Participants Sociodemographic characteristics.

Variable	Category	n (%)
PrEP persistence Status	Not persistent	18 (56%)
	Persistent	14 (44%)
Marital Status	Single	21 (66%)
	Married to women	6 (19%)
	Cohabiting with male partner	5 (16%)
Sexual position identity	Top	9 (28%)
	Versatile	8 (26)%
	Bottom	15 (46%)
Age Group	18–24 years	12 (38%)
	25–34 years	15 (47%)
	35+ years	5 (16%)
Education Level	Primary or below	13 (41%)
	Secondary	15 (47%)
	Tertiary	4 (13%)
Occupation	Motorcycle driver (*bodaboda*)	9 (28%)
	Small business/vendor	11 (34%)
	Student	6 (19%)
	Unemployed	6 (19%)

## Data Availability

The data used in this analysis are available upon reasonable request. Contact details: Ms. Faithness Kiondo; E-mail: kiondofaithness@gmail.com.

## Abbreviations

The following abbreviations are used in this manuscript:

AIDS	Acquired immune deficiency syndrome
ARV/ARVs	Antiretroviral drug(s)
COVID-19	Coronavirus disease 2019
DOCEHTA	Strengthening Doctoral Education for Health in Tanzania
DRC	Democratic Republic of the Congo
HIV	Human immunodeficiency virus
IRB	Institutional Review Board
MUHAS	Muhimbili University of Health and Allied Sciences
NORAD	Norwegian Programme for Capacity Development in Higher Education and Research for Development
PrEP	Pre-exposure prophylaxis
REC	Research Ethics Committee
UNAIDS	Joint United Nations Programme on HIV/AIDS
WHO	World Health Organisation

## References

[1] Joint United Nations (2025). Programme on HIV/AIDS (UNAIDS). AIDS, Crisis and the Power to Transform: UNAIDS Global AIDS Update 2025.

[2] The Global Fund (2025). Results Report 2025.

[3] World Health Organization (2024). Global Health Observatory Data: HIV/AIDS.

[4] Oldenburg C.E., Perez-Brumer A.G., Reisner S.L., Mimiaga M.J., Hatzenbuehler M.L., Bärnighausen T. (2018). Human rights protections and HIV prevalence among MSM who sell sex: Cross-country comparisons from a systematic review and meta-analysis. Glob. Public Health.

[5] Jin H., Restar A.J., Beyrer C., Tang W. (2021). Epidemiological conditions of HIV among key populations in Nairobi, Kenya: Implications for HIV prevention, treatment and care. PLoS ONE.

[6] Schweitzer A.M., Dišković A., Krongauz V., Newman J., Tomažič J., Yancheva N. (2023). Addressing HIV stigma in healthcare, community, and legislative settings in Central and Eastern Europe. AIDS Res. Ther..

[7] Stoebenau K., Muchanga G., Ahmad S.S., Bwalya C., Mwale M., Toussaint S., Maambo C., Peters C.J., Baumhart C., Mwango L.K. (2024). Barriers and facilitators to uptake and persistence on prep among key populations in Southern Province, Zambia: A thematic analysis. BMC Public Health.

[8] Bandura A. (1986). Social Foundations of Thought and Action: A Social Cognitive Theory.

[9] Donnell D., Baeten J.M., Bumpus N.N., Brantley J., Bangsberg D.R., Haberer J.E., Mujugira A., Mugo N., Ndase P., Hendrix C. (2014). HIV protective efficacy and correlates of tenofovir blood concentrations in a clinical trial of PrEP for HIV prevention. J. Acquir. Immune Defic. Syndr..

[10] Nicol M.R., Adams J.L., Kashuba A.D. (2013). HIV PrEP trials: The road to success. Clin. Investig..

[11] Dimitrov D.T., Mâsse B.R., Donnell D. (2016). PrEP adherence patterns strongly affect individual HIV risk and observed efficacy in randomized clinical trials. J. Acquir. Immune Defic. Syndr..

[12] Chapin-Bardales J., Haaland R., Martin A., Holder A., Butts V.A., Sionean C., Sey E.K., Brady K.A., Raymond H.F., Opoku J. (2023). HIV Pre-exposure Prophylaxis Persistence and Adherence Among Men Who Have Sex with Men in Four US Cities. J. Acquir. Immune Defic Syndr..

[13] Shangase N., Jiyane A., Buthelezi F., O’Connor C., Brown B., Rees K. (2025). Pre-exposure prophylaxis persistence at two sites in an integrated primary health care programme in South Africa. Front. Public Health.

[14] Rao A., Mhlophe H., Pretorius A., Mcingana M., Mcloughlin J., Shipp L., Baral S., Hausler H., Schwartz S., Lesko C. (2023). Effect of implementation strategies on pre-exposure prophylaxis persistence among female sex workers in South Africa: An interrupted time series study. Lancet HIV.

[15] Ongolly K., Dolla A., Ngure K., Irungu E.M., Odoyo J., Wamoni E., Peebles K., Mugwanya K., Mugo N.R., Bukusi E.A. (2021). “I Just Decided to Stop:” Understanding PrEP Discontinuation Among Individuals Initiating PrEP in HIV Care Centers in Kenya. J. Acquir. Immune Defic. Syndr..

[16] Pico-Espinosa O.J., Hull M., MacPherson P., Grace D., Gaspar M., Lachowsky N., Mohammed S., Demers J., Kilduff M., Truong R. (2022). PrEP-related stigma and PrEP use among gay, bisexual and other men who have sex with men in Ontario and British Columbia, Canada. AIDS Res. Ther..

[17] Davoudpour S., Phillips G.L., Serrano P.A., French A.L., Hosek S.G. (2024). A Memo on Factors Associated with Perception of Stigma Attached to PrEP: Evidence from the Keeping It LITE Study. Sexes.

[18] Psaros C., Hill-Rorie J., Quint M., Horvitz C., Dormitzer J., Biello K.B., Krakower D.S., Safren S.A., Mimiaga M.J., Sullivan P. (2024). A qualitative exploration of how to support PrEP adherence among young men who have sex with men. AIDS Care.

[19] Link B.G., Phelan J.C. (2001). Conceptualizing stigma. Annu. Rev. Sociol..

[20] Dubov A., Galbo P., Altice F.L., Fraenkel L. (2018). Stigma and Shame Experiences by MSM Who Take PrEP for HIV Prevention: A Qualitative Study. Am. J. Mens. Health.

[21] Tuan L.A., Pham L.Q., Khuyen T.T., Hao B.M., Hoa N.T.P., Vu K.-D., Lien T.H.M., Duyen P.T.T., Phan H.T., Giang L.M. (2025). Facilitators and Barriers to Hiv Pre-Exposure Prophylaxis Adherence and Retention Among Young Men Who have Sex With Men: A Meta-Ethnographic Scoping Review. AIDS Behav..

[22] Rosengren A.L., Lelutiu-Weinberger C., Woodhouse E.W., Sandanapitchai P., Hightow-Weidman L.B. (2021). A Scoping Review of HIV Pre-exposure Prophylaxis Stigma and Implications for Stigma-Reduction Interventions for Men and Transwomen Who Have Sex with Men. AIDS Behav..

[23] Earnshaw V.A., Chaudoir S.R. (2009). From conceptualizing to measuring HIV stigma: A review of HIV stigma mechanism measures. AIDS Behav..

[24] Tisdell E.J., Merriam S.B., Stuckey-Peyrot H.L. (2025). Qualitative Research: A Guide to Design and Implementation.

[25] Braun V., Clarke V. (2022). Toward good practice in thematic analysis: Avoiding common problems and be(com)ing a knowing researcher. Int. J. Transgend Health.

[26] Lincoln Y., Guba E.G. (1985). Naturalistic Inquiry.

[27] Goffman E. (1963). Stigma: Notes on the Management of Spoiled Identity.

[28] Moen K., Aggleton P., Leshabari M.T., Middelthon A.L. (2014). Gays, Guys, and Mchicha Mwiba: Same-Sex Relations and Subjectivities in Dar es Salaam. J. Homosex..

[29] Shio J., Moyer E. (2020). Navigating norms of masculinity: Tactical gender performances among gay men in Dar Es Salaam, Tanzania. Gend. Place Cult..

[30] Pachankis J.E., Bränström R. (2019). How many sexual minorities are hidden? Projecting the size of the global closet with implications for policy and public health. PLoS ONE.

[31] Pachankis J.E., Hatzenbuehler M.L., Hickson F., Weatherburn P., Berg R.C., Marcus U., Schmidt A.J. (2015). Hidden from health: Structural stigma, sexual orientation concealment, and HIV across 38 countries in the European MSM Internet Survey. AIDS.

[32] Gios L., Mirandola M., Sherriff N., Toskin I., Blondeel K., Dias S., Staneková D., Folch C., Schink S.B., Nöstlinger C. (2021). Being in the Closet. Correlates of Outness Among MSM in 13 European Cities. J. Homosex..

[33] Kerr J., Ayangeakaa S., Bullock N.A., Burton K., Combs R., Harris L., Sterrett-Hong E., Rozema I., Sears J., Northington T. (2022). A qualitative exploration of various stigmas impacting HIV pre-exposure prophylaxis (PrEP) uptake among African American young adults. Fam. Community Health.

[34] Lichtwarck H.O., Massawe E.P., Mmbaga E.J., Moen K. (2024). What can PrEP do for female sex workers? Unpacking the “effectosphere” of biomedical HIV prevention in Dar es Salaam. Soc. Sci. Med..

[35] Adeagbo O., Harrison S., Qiao S., Li X. (2021). Pre-exposure prophylaxis (PrEP) uptake among black men who have sex with men (BMSM) in the Southern US. Int. J. Environ. Res. Public Health.

[36] Alie M.S., Abebe G.F., Negesse Y., Adugna A., Girma D. (2024). Vaccine hesitancy in context of COVID-19 in East Africa: Systematic review and meta-analysis. BMC Public Health.

[37] Ochola E.A. (2023). Vaccine hesitancy in Sub-Saharan Africa in the Context of COVID-19 vaccination exercise: A systematic review. Diseases.

[38] Graybill L.A., McKay C.N., Wang J., Sam-Agudu N.A., Yotebieng M., Saidi F., Bekker L.G., Shook-Sa B.E., Chi B.H., Rosenberg N.E. (2025). Characterizing populations prioritized for PrEP in 19 African countries: A review of national guidance. J. Int. AIDS Soc..

[39] Kigombola A., Lyimo J., Mizinduko M., Mkembela D., Maziku E., Kafura W., Maghimbi A., Musanhu C., Nsubuga P., Yoti Z. (2023). Low engagement of key populations in HIV health services in Tanzania: Analysis of community, legal and policy factors. Pan Afr. Med. J..

[40] Kiondo F., Metta E., Mmbaga E.J., Leshabari M.T., Swai C., Mbotwa C.H., Moen K. (2025). Retention in HIV Pre-Exposure Prophylaxis Among Men Who Have Sex with Men in Tanga, Tanzania. HIV AIDS.

[41] Zotova N., Shongo A., Lelo P., Mbonze N., Kaba D., Ntangu P., Shi Q., Adedimeji A., Anastos K., Yotebieng M. (2025). Attrition from Care and Barriers to PrEP Use Among Key Populations in Kinshasa, DRC: A Multiple Methods Study. AIDS Behav..

[42] Golub S.A., Fikslin R.A., Goldberg M.H., Peña S.M., Radix A. (2019). Predictors of PrEP Uptake Among Patients with Equivalent Access. AIDS Behav..

[43] Golub S.A., Starbuck L., Fikslin R., Gamarel K.E. (2023). Psychometric Evaluation and Predictive Validity of an Adapted Adherence Self-Efficacy Scale for PrEP. AIDS Behav..

[44] Whiteley L., Craker L., Sun S., Tarantino N., Hershkowitz D., Moskowitz J., Arnold T., Haubrick K., Olsen E., Mena L. (2021). Factors associated with PrEP adherence among MSM living in Jackson, Mississippi. J. HIV AIDS Soc. Serv..

[45] Bauermeister J.A., Downs J.S., Krakower D.S. (2020). PrEP product acceptability and dual process decision-making among men who have sex with men. Curr. HIV/AIDS Rep..

[46] Ireland M.E., Schwartz H.A., Chen Q., Ungar L.H., Albarracín D. (2015). Future-oriented tweets predict lower county-level HIV prevalence in the United States. Health Psychol..

[47] Murphy L., Dockray S. (2018). The consideration of future consequences and health behaviour: A meta-analysis. Health Psychol. Rev..

[48] Gichane M.W., Velloza J., Hosek S., Beauchamp G., Anderson P., Delany-Moretlwe S., Celum C. (2025). Hoping to Adhere? Examining the Relationship Between Hope and Pre-exposure Prophylaxis Willingness, Adherence, and Persistence Among Young Women in South Africa and Zimbabwe (HPTN 082). AIDS Behav..

[49] Brooks R.A., Cabral A., Nieto O., Fehrenbacher A., Landrian A. (2019). Experiences of Pre-Exposure Prophylaxis Stigma, Social Support, and Information Dissemination Among Black and Latina Transgender Women Who Are Using Pre-Exposure Prophylaxis. Transgend. Health.

[50] Sheira L.A., Kwena Z.A., Ayieko B., Charlebois E.D., Agot K., Gutin S.A., Lewis-Kulzer J., Olugo P., Gandhi M., Bukusi E.A. (2025). The effect of a social network-based intervention on adherence to HIV preexposure prophylaxis and HIV viral suppression among Kenyan fishermen. AIDS.

[51] Rugira E., Biracyaza E., Umubyeyi A. (2023). Uptake and Persistence on HIV Pre-Exposure Prophylaxis Among Female Sex Workers and Men Having Sex with Men in Kigali, Rwanda: A Retrospective Cross-Sectional Study Design. Patient Prefer. Adherence.

